# Perinatal changes in ventricular mechanics in transposition of the great arteries: a prospective longitudinal speckle-tracking echocardiography study

**DOI:** 10.3389/fcvm.2026.1865975

**Published:** 2026-08-19

**Authors:** Andreea Cerghit-Paler, Mihaela Iancu, Dorottya Gabor-Miklosi, Amalia Făgărășan, Iolanda Muntean, Carmen-Corina Șuteu, Daniela Toma, Claudiu Mărginean, Liliana Gozar

**Affiliations:** 1 Department of Pediatrics III, George Emil Palade University of Medicine, Pharmacy, Science, and Technology of Targu Mures, Târgu Mureș, Romania; 2 Department of Medical Informatics and Biostatistics, “Iuliu Hațieganu” University of Medicine and Pharmacy, Biostatistics and Medical Informatics, Fundamental Disciplines, Cluj-Napoca, Romania; 3 Pediatric Cardiology, Emergency Institute of Cardiovascular Diseases and Transplantation, Târgu-Mureș, Romania; 4 Department of Obstetrics and Gynecology, George Emil Palade University of Medicine, Pharmacy, Science, and Technology Targu Mures, Târgu-Mureș, Romania

**Keywords:** fetal echocardiography, global longitudinal strain, myocardial strain, neonatal cardiac function, speckle-tracking echocardiography, transposition of the great arteries

## Abstract

**Background:**

Transposition of the great arteries (TGA) is characterized by parallel circulation dependent on intercirculatory mixing. The impact of perinatal hemodynamic changes on myocardial mechanics remains poorly understood. The main objective of the study was to evaluate perinatal changes in ventricular strain in TGA.

**Methods:**

The current prospective longitudinal study included 79 fetuses, of whom 72 were included after applying predefined exclusion criteria (19 with simple TGA and 53 healthy controls). Speckle-tracking echocardiography was used to assess segmental and global ventricular strain. Differences in temporal changes between groups were analyzed using linear regression models with robust standard errors clustered at the subject level. Estimated marginal means (EMMS) with 95% confidence intervals (CI) were derived to compare groups at each timepoint (T0: fetal evaluation; T1:neonatal evaluation).

**Results:**

Fetal myocardial deformation parameters did not differ significantly between the TGA and control groups (*p* > 0.05, corrected *p* > 0.05). Between-group differences at the neonatal assessment are nominally significant for the basal, medial, and apical interventricular septal segments and for right ventricle (RV) peak longitudinal strain from 4 chamber view (*p* < 0.05). After Benjamini-Hochberg correction, however, only the medial interventricular septal strain remained statistically significant (*p*-corrected = 0.0242). Additionally, RV end-systolic volume measured at neonatal time point was significantly increased in neonates with TGA compared to healthy controls [EMMS: 2.50 mL (95% CI: 2.25–2.76) vs. 1.30 mL (95% CI: 1.19–1.41)] reflecting altered loading conditions. Although RV peak longitudinal strain from 4 chamber view significantly differed between neonates requiring balloon atrial septostomy and those who did not (*p* = 0.007), no significant discriminatory ability was observed (AUC = 0.73, 95% CI: 0.46–1). As secondary outcomes, these parameters were not subject to correction for multiple testing.

**Conclusions:**

In this cohort of prenatally diagnosed simple TGA, fetal myocardial deformation parameters did not differ significantly from those of healthy controls, whereas load-sensitive regional differences in interventricular septal and RV deformation identified as nominal associations, though not all retained significance after correction for multiple testing, became apparent after birth. These findings suggest that postnatal changes in loading conditions may reveal regional mechanical differences that are not evident during fetal life.

## 1 Introduction

Simple transposition of the great arteries (TGA) is a cardiac malformation defined by atrioventricular concordance and ventriculoarterial discordance, with an intact interventricular septum and no other associated cardiac anomalies. It accounts for approximately 10% of neonatal cyanotic congenital heart defects and represents one of the most common critical congenital heart diseases (1, 2). The pathophysiology of TGA is defined by the presence of two parallel circulations, incompatible with extrauterine life in the absence of adequate intra- or extracardiac communications, such as patent foramen ovale (PFO) and patent ductus arteriosus (PDA). Although fetal hemodynamics allow survival in utero, the neonatal circulatory transition transforms TGA into a major cardiac emergency. Perinatal circulatory changes can be associated with restriction of the PFO and/or PDA (3–6), and with redistribution of blood flow (7, 8). These alterations can lead to severe hypoxemia, rapid circulatory compromise, and significant early morbidity after birth (9). Moreover, the abrupt postnatal transition from fetal parallel circulation to neonatal physiology exposes the ventricles, particularly the systemic right ventricle and interventricular septum, to rapidly changing loading conditions that may lead to regional changes in myocardial deformation and impaired perioperative adaptation.

Hemodynamic changes occurring during late gestation and early neonatal life are substantial but only partially detectable by conventional echocardiographic techniques, both in healthy fetuses and in those with TGA (10, 11). Variations in ventricular loading conditions, particularly changes in afterload and preload, may obscure early changes in myocardial deformation when assessed using standard echocardiographic parameters. Although current imaging techniques, such as tissue Doppler imaging (TDI) and speckle-tracking echocardiography (STE), have demonstrated superior sensitivity in detecting subtle and subclinical changes in fetal and neonatal myocardial strain (12–15), data on the evaluation of myocardial deformation in fetuses with TGA remain limited. This gap in knowledge is primarily related to the lack of studies conducted during the critical period of hemodynamic transformations, as well as the fact that current studies are based on retrospective research designs and the predominant use of conventional echocardiographic techniques, which have led to inconsistent results. Compared with conventional echocardiographic parameters, speckle-tracking echocardiography provides a quantitative and angle-independent assessment of myocardial deformation. STE allows evaluation of both global and segmental ventricular function and may detect subclinical myocardial impairment even when ejection fraction and standard echocardiographic indices remain preserved (12–15).

Although previous prospective studies have described morphometric, systolic, diastolic and hemodynamic changes during the fetal-to-neonatal transition in TGA, information regarding regional myocardial deformation assessed by speckle-tracking echocardiography remains limited. Therefore, the present study aimed to characterize global and regional biventricular strain patterns throughout this transition and to determine whether strain analysis provides additional insights beyond conventional echocardiographic parameters.

The primary objective was to evaluate perinatal changes in ventricular myocardial deformation assessed by strain analysis in TGA and control groups. Secondary objectives included comparison of conventional echocardiographic parameters between TGA and control groups, as well as exploratory analysis of differences between neonates with TGA requiring balloon atrial septostomy and those who did not.

## 2 Material and methods

### 2.1 Study design and population

We conducted a prospective longitudinal comparative study between September 28, 2023 and February 27, 2025, within the Pediatric Cardiology Clinic of the Emergency Institute for Cardiovascular Diseases and Cardiac Transplantation in Târgu Mureș.

Inclusion criteria: fetuses diagnosed intrauterine with simple TGA in the third trimester of pregnancy and who underwent echocardiographic evaluation in the early neonatal period. The control group included fetuses in the last trimester of pregnancy, without obvious cardiac or extracardiac pathology, and who underwent echocardiographic evaluation in the first days of life.

**Figure 1:**
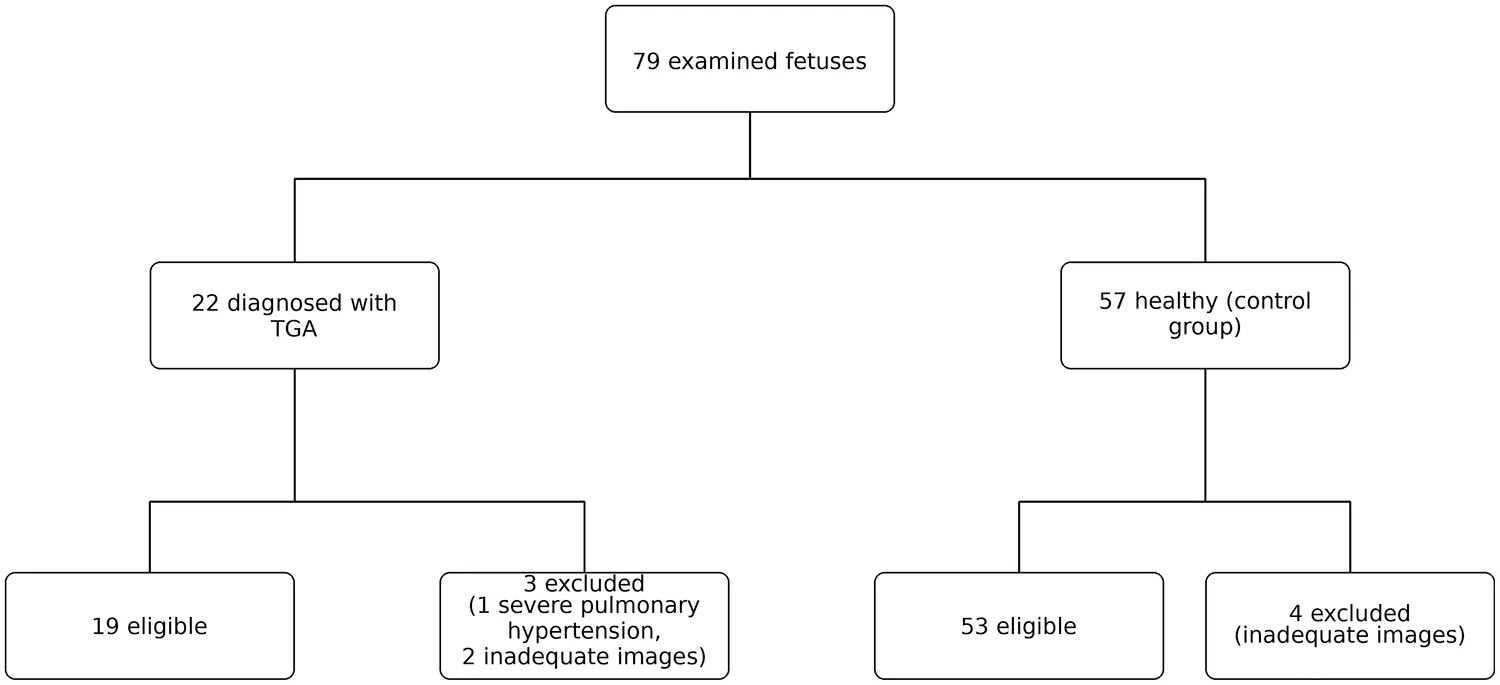
Study flow-chart. TGA, Transposition of the great arteries.

Exclusion criteria were prospectively predefined and included complex TGA, inadequate echocardiographic image quality for strain analysis, associated cardiac or extracardiac malformations and severe pulmonary hypertension. Accordingly, one newborn with severe pulmonary hypertension, which required the administration of nitric oxide, was excluded based on this predefined clinical criterion, as well as 6 fetuses whose echocardiographic images were of insufficient quality for strain analyses. Thus, 72 of the 79 participants were included in the final analysis (19 with TGA and 53 controls), after exclusion criteria were applied at different stages of the study. (Figure 1).

Participants were prospectively followed according to a predefined protocol. Serial evaluations were conducted from the prenatal period through the early neonatal period. Fetuses diagnosed intrauterine with simple TGA were evaluated by complex echocardiography in the third trimester of pregnancy. After birth, the newborns underwent a standardized echocardiographic evaluation in the first days of life, before performing any invasive therapeutic intervention, especially balloon atrial septostomy, where indicated. This approach allowed the analysis of myocardial deformation in a relatively stable subgroup of prenatally diagnosed simple TGA before invasive therapeutic interventions whenever clinically feasible. Consequently, the findings should be interpreted within this selected clinical context and are not intended to represent the full spectrum of TGA presentation. Similarly, fetuses included in the control group were evaluated echocardiographically in the last trimester of pregnancy, in the absence of any identified cardiac or extracardiac pathology, and subsequently re-evaluated in the early neonatal period, in the first days of life. This longitudinal follow-up strategy allowed direct comparison of cardiac functional parameters between the study group and the control group, both in the intrauterine period and immediately postnatally.

Demographic and clinical data monitored in the study were gestational age (GA) at the time of complex echocardiography, fetal growth: femur length (FL) and biparietal diameter (BPD), sex, GA at birth, age in hours of the newborn at the time of complex echocardiography evaluation, birth weight, length at birth, APGAR score, heart rate, oxygen saturation at birth, need for initiation of PEV with prostaglandin, need for Rashkind septostomy.

Fetal and neonatal echocardiographies were performed by a pediatric cardiologist experienced in fetal cardiology, using a Philips Epiq 7 ultrasound machine. Fetal echocardiographic examinations were performed during the third trimester of pregnancy, between 33 and 38 weeks of gestation in the TGA group and between 30 and 39 weeks in the control group. A complete echocardiographic evaluation was performed, in order to establish the diagnosis of simple TGA. For each fetus and newborn, a minimum of three two-dimensional echocardiographic acquisitions from the apical four-chamber section were obtained, with a frame rate greater than 80 Hz. The images were subsequently stored in Digital Imaging and Communications in Medicine (DICOM) format. Fetal echocardiographic examinations were performed during the third trimester of pregnancy, within the gestational age range reported in Table 1. Neonatal echocardiography was performed in the early postnatal period, before any invasive therapeutic intervention, within the first 24–72 hours after birth whenever clinically feasible.

**Table 1:** Demographic and anthropometric characteristics of the neonates.

Variables	Control group (*n*_1_ = 53)	TGA group (*n*_2_ = 19)	*p*-value
Gestational age at enrolment (weeks)	35 (34, 36) Range: 30–39	36 (36, 37) Range: 33–38	0.0020*
Gestational age at delivery (weeks)	40 (38, 40) Range: 37–42	39 (39, 40) Range: 35–40	0.9198
Time between assessments, weeks	4 (3, 6)	3 (2, 3)	0.0076*
Age, hours, median (IQR)	30 (25, 50)	23 (22, 38)	0.1126
Male Sex (%)	32 (60.4)	16 (84.2)	0.0587
FL (%)	34.70 (28.50, 44.20)	34.20 (32.55, 42.15)	0.7688
BPD (%)	61.60 (13.24)	64.14 (10.93)	0.4565
Heart rate at birth (beats/min)	134.11 (7.20)	138 (11.31)	0.1747
SaO2 at birth (%)	100 (99, 100)	82 (80, 85)	<0.0001*
Birth weight (kg)	3.43 (0.34)	3.35 (0.30)	0.3761
Birth height (cm)	52.81 (2.02)	53.05 (1.93)	0.6527
APGAR score	10 (9, 10)	9 (8, 9)	<0.0001*
Rashkind_septostomy at birth	NA	12 (63.16)	NA
Prostaglandin_infusion at birth	NA	17 (89.47)	NA

FL, femur length; BPD, biparietal diameter; TGA, Transposition of the great arteries; SaO2, oxygen saturation; IQR, interquartile range defined as (Q1, Q3) where Q1 denoted the first quartile, Q3 denoted the third quartile; NA, not applicable.

* Significant result: *p* < 0.05.

### 2.2 Speckle-tracking analysis

Speckle-tracking analysis was subsequently performed offline by a single operator who was blinded to group allocation. From the stored DICOM images, the highest quality ones were selected and analyzed offline, using Philips QLAB 15.0 software (material number 453562090801), with Auto Cardiac Motion Quantification (aCMQ) function and autostrain function for the left ventricle (LV) and the right ventricle (RV). In the fetal setting, using the aCMQ function, in the absence of a fetal ECG, the cardiac cycle was manually identified by the operator, based on the mechanical movements of the mitral valve (its complete closure defining the end-diastole). Following the manual identification of the cardiac cycle, the software calculated the heart rate, which was compared with the value obtained by Doppler examination.

After establishing the cardiac cycle, the operator selected three endocardial points in a predefined order. Initially, the base of the interventricular septum was marked, followed by the lateral edge of the mitral annulus, and the third point was represented by the apex. Based on these three points, the software automatically generated the endocardial contour, which was subsequently verified and manually adjusted when necessary.

**Figure 2:**
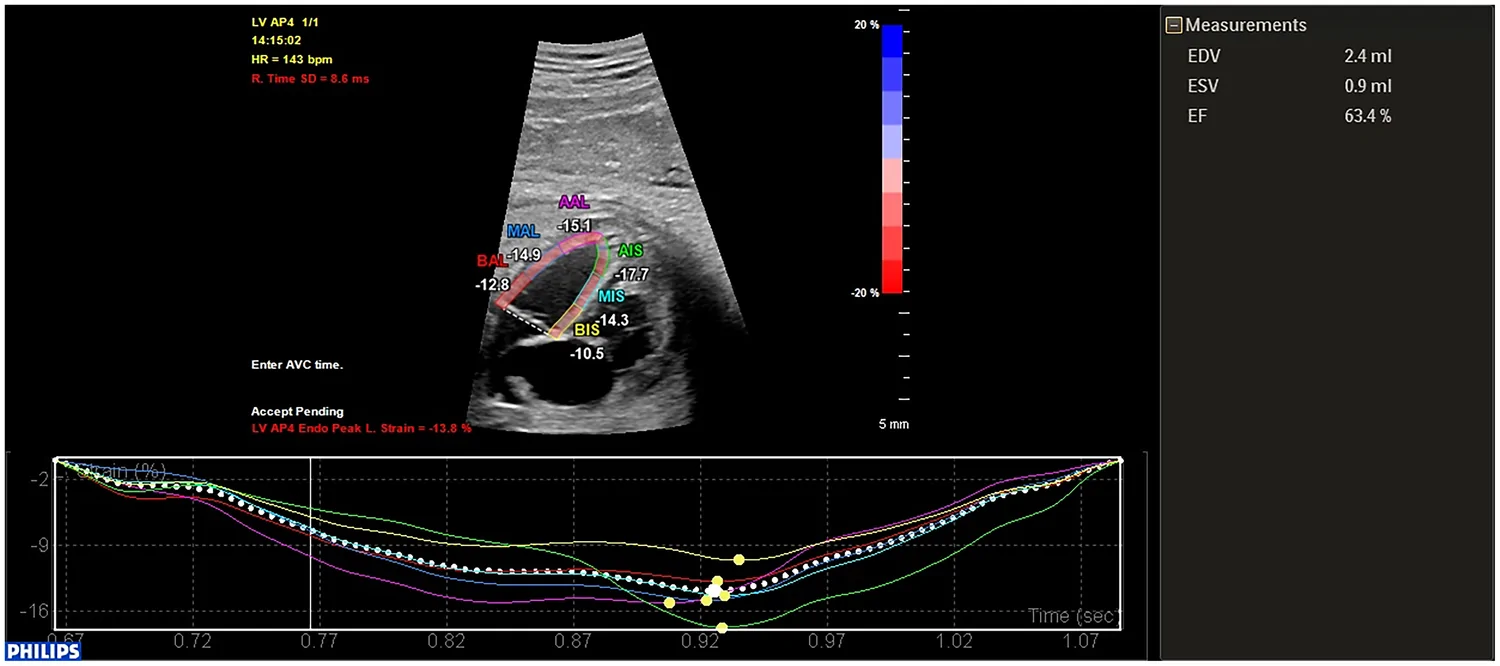
Fetal left ventricle speckle tracking analysis. LV, left ventricle; AP4, 4 chamber view; HR, heart rate; SD, standard deviation; EDV, end-diastolic volume; ESV, end-systolic volume; EF, ejection fraction; LV AP4 Endo Peak L. Strain, left ventricle peak longitudinal strain from 4 chamber view; BAL, basal lateral wall; MAL, medial lateral wall; AAL, apical lateral wall; BIS, basal interventricular septum; MIS, medial interventricular septum; AIS, apical interventricular septum.

Speckle tracking parameters obtained included regional strain (basal, medial and apical interventricular septum; basal, medial and apical LV; basal, medial and apical RV), global biventricular strain: RV peak longitudinal strain from 4 chamber view (RVAP4pLS) and LV peak longitudinal strain from 4 chamber view (LVAP4pLS); biventricular volumes: end-diastolic volume (EDV) and end-systolic volume (ESV), biventricular ejection fraction (EF) and biventricular R time (Figure 2). These parameters were included in the database and statistically analyzed.

**Figure 3:**
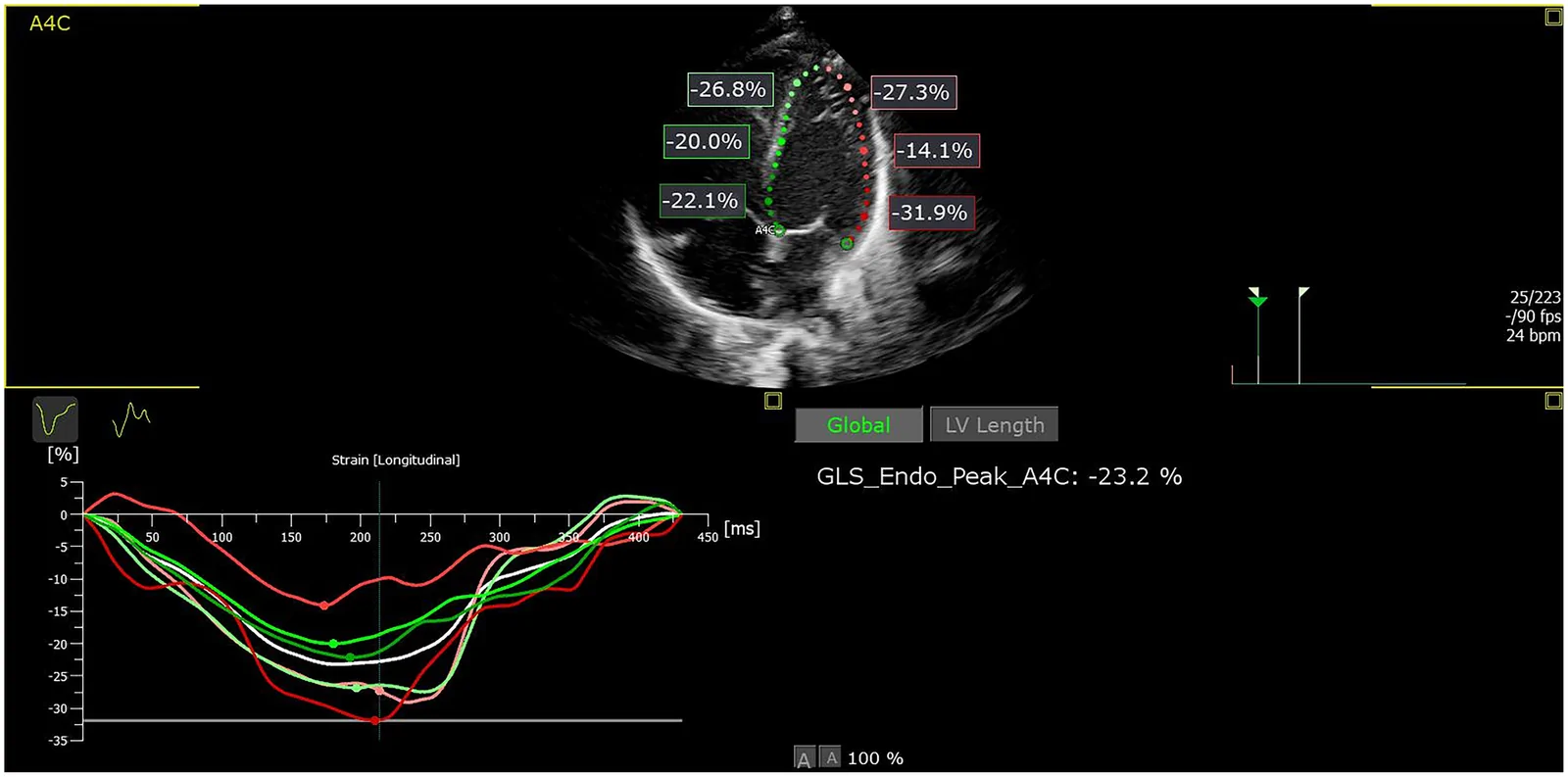
Neonatal autostrain left ventricle evaluation. GLS_Endo_Peak_A4C: left ventricle peak longitudinal strain from apical 4 chamber view.

In the early neonatal period, aCMQ was maintained for comparative purposes and complemented by the AutoStrain function for the LV (Figure 3) and RV. With autostrain function, cardiac cycle timing was established based on M-mode imaging, allowing precise identification of end-diastolic and end-systolic frames. Automated endocardial border detection was subsequently performed, followed by systematic evaluation of tracking quality using the tracking revision function. In cases of inadequate tracking, manual adjustments of the endocardial contours were undertaken to optimize the region of interest and ensure accurate speckle-tracking–derived strain measurements.

Ventricular volume assessment was performed using the automated Cardiac Motion Quantification (aCMQ) function implemented in Philips QLAB 15.0 for both fetal and neonatal examinations. Following endocardial contour definition and manual adjustment when necessary, the software performed frame-by-frame tracking throughout the cardiac cycle and automatically derived end-diastolic and end-systolic ventricular volumes. In the neonatal examinations, AutoStrain LV and AutoStrain RV were additionally used for myocardial deformation analysis, whereas ventricular volumes were derived using aCMQ. For the right ventricle, the specific geometric assumptions underlying volume calculation are incorporated within the software algorithm and are not explicitly provided to the operator. Therefore, RV volumes were considered software-derived estimates, and specific validation of this approach for fetal and neonatal right ventricular volumetry is limited. Ventricular volumes were expressed in milliliters (mL) and were not indexed to body surface area or another measure of body size.

Global longitudinal strain (GLS) was calculated as the average of segmental longitudinal strain values and was used as an index of global ventricular function. GLS is recognized as a sensitive marker of subclinical changes in myocardial deformation in fetal and pediatric population and may detect early impairment in ventricular performance even in the presence of preserved ejection fraction, as supported by current reviews, International Society of Ultrasound in Obstetrics and Gynecology (ISUOG) and American Society of Echocardiography (ASE) society recommendations (16–19).

The regional and global biventricular strain parameters were considered the primary outcomes of the current study while conventional echocardiographic parameters and the need for Rashkind septostomy were considered secondary outcomes.

### 2.3 Ethics

The entire research protocol was conducted in accordance with the principles stated in the declaration of Helsinki. A signed, informed consent detailing the methodology of the study was provided for each pregnant woman enrolled in the study. Those who refused to provide written consent for were excluded. The present research was approved by the Ethical Committee of the Emergency Institute of Cardiovascular Disease and Heart Transplantation from Targu Mures and “George Emil Palade” University of Medicine and Pharmacy Targu-Mures 1276/25.02.2021.

### 2.4 Statistical analysis

Distributions of quantitative demographic and clinical characteristics in each of the studied groups were assessed using descriptive statistics, quantile-quantile plots and Shapiro–Wilk test. All variables with a normal probability distribution were summarized using arithmetic and sample standard deviation (SD) while variables departing from normal probability distribution were described by median and interquartile range (IQR). Comparisons of demographic and anthropometric characteristics between the studied groups were performed using Student t-test or Mann–Whitney *U*-test.

Qualitative characteristics were described by counts (absolute frequencies) and percentages (relative frequencies). Between-group differences in qualitative characteristics were tested using Chi-square test or Fisher's exact test.

Differences in change over time of strain parameters between TGA and control groups were tested using linear regression with robust standard errors clustered at the patient level, to account for within-patient correlation arising from repeated measurements. As the two evaluations of the strain parameters were not performed at fixed timepoints—the second evaluation being conducted at birth—gestational age at enrolment and gestational age at birth were considered as covariates in all regression models (to account for the variability in developmental stage across neonates). Each model tested included timepoint (fetal vs. neonatal), group (Group TGA vs. Control), their interaction term as predictors and gestational age at enrolment, gestational age at birth, age (hours) and sex as covariates. Robust standard errors were computed using “sandwich” R package (20, 21).

Differences in the distribution of fetal myocardial strain parameters between neonates with TGA who required septostomy and those who did not were evaluated while controlling for potential confounders (gestational age at enrolment) were tested using the analysis for covariance (ANCOVA).

Estimated marginal means (EMMS) with 95% confidence intervals (95% CI) were derived from all the tested linear models and represented the model-predicted mean values. EMMS are shown to facilitate the interpretation of adjusted between-group at each timepoint and were calculated using “emmeans” R package (22).

All descriptive and inferential statistics were performed using R statistical software (version 4.5.2, R Foundation for Statistical Computing, Vienna, Austria). Statistical significance for all two-sided tests was set at *p* < 0.05.

For comparisons across strain parameters, correction for multiple comparisons was performed using Benjamini-Hochberg (BH) procedure to control the false discovery rate. The BH correction was applied specifically to the within-group temporal (perinatal) changes in strain parameters (left ventricle, interventricular and right ventricle global and segmental strain measures) and between-group comparisons at each time point. The BH procedure was applied separately to two family of tests: the 11 within -group temporal comparisons in each group and the family of 22 between-group comparisons across the two time points. Group-by-time interaction tests were reported as nominal p values without correction for multiple testing. Estimated significance levels (p-values) derived from BH procedure were denoted as *p*-corrected. Comparisons of the conventional echocardiographic parameters and the need for Rashkind septostomy were considered exploratory analysis and no formal correction for multiple testing was applied.

## 3 Results

Patients' characteristics are presented in Table 1. Compared with healthy neonates, neonates with TGA showed similar distributions of age, sex and BPD (*p* > 0.05) except for gestational age at inclusion (*p* = 0.004). The inter-assessment time interval was 4 weeks (IQR: 3–6) in the control group and 3 weeks (IQR: 2–3) in the TGA group.

### 3.1 Differences in left ventricle, interventricular and right ventricle strain measures

Differences in the mean values of segmental and longitudinal strain parameters were estimated using linear regression models with robust standard errors, accounting for within-patient correlations (Table 2). For the interventricular medial segmental strain measurements, a significant group ⨯ time interaction effect was observed (*p* = 0.0134). Results of *post-hoc* analysis revealed significant main effect for time was found for LV basal (*p* < 0.001 in the TGA group and *p* = 0.0008 in the control group), LV apical (*p* = 0.0017 in the TGA group and *p* = 0.0058 in the control group), interventricular basal segment (*p* = 0.6664 in TGA vs. *p* = 0.006 in controls), interventricular apical segment (*p* < 0.0001 in both groups), LVAP4pLS (*p* = 0.0621 in the TGA group vs. *p* = 0.0002 in controls), RV basal, medial and apical segment (*p* < 0.0001 in both groups), RV longitudinal strain RVAP4pLS (*p* = 0.0059 in the TGA groups vs. *p* < 0.0001 in controls). After corrections for multiple comparisons, significant perinatal changes were observed for LV basal (*p*-corrected = 0.0002 in the TGA group vs. 0.0014 in the control group), LV apical (*p*-corrected = 0.003 in the TGA group vs. 0.008 in the control group), interventricular basal segment (*p*-corrected = 0.0004 in controls), interventricular apical segment (*p* = 0.0002 in both groups), LVAP4pLS (*p*-corrected = 0.0002 in controls), RV basal, medial and apical segment (*p*-corrected = 0.0002 in both groups) and RV longitudinal strain RVAP4pLS (*p*-corrected = 0.008 in the TGA group vs. *p*-corrected = 0.0002 in controls).

**Table 2:** Differences in estimated marginal means (EMMS) of left ventricle, interventricular and right ventricle strain parameters between studied groups.

Time point	EMMS (95% CI) inControl group (*n*_1_ = 53)	EMMS (95% CI) in TGA group (*n*_2_ = 19)	Adjusted difference	*p*-value^a^	*p*-corrected^b^
LV_Basal (%)					
T0	−16.9 (−18.0, −15.8)	−15.7 (−17.6, −13.9)	−1.15	0.3091	0.5231
T1	−28.3 (−30.9, −25.8)	−23.0 (−26.3, −19.8)	−5.32	0.0103*	0.1133
LV_Medial (%)					
T0	−15.9 (−17.1, −14.8)	−16.3 (−18.3, −14.3)	0.36	0.7530	0.9203
T1	−15.2 (−16.3, −14.0)	−14.9 (−16.9, −12.9)	−0.28	0.8100	0.9379
LV_Apical (%)					
T0	−15.7 (−17.2, −14.3)	−15.7 (−18.3, −13.2)	0.001	0.9997	0.9997
T1	−19.0 (−20.4, −17.5)	−20.0 (−22.5, −17.5)	1.03	0.4849	0.6938
Inter_V_Basal (%)					
T0	−15.7 (−16.8, −14.6)	−14.7 (−16.7, −12.8)	−0.94	0.4156	0.6531
T1	−17.9 (−19.0, −16.8)	−15.4 (−17.4, −13.4)	−2.49	0.0321*	0.1434
Inter_V_Medial (%)					
T0	−15.9 (−16.9, −14.9)	−15.9 (−17.7, −14.1)	0.03	0.9792	0.9997
T1	−20.0 (−21.0, −19.0)	−16.6 (−18.3, −14.8)	−3.43	0.0011*	0.0242*
Inter_V_Apical (%)					
T0	−15.9 (−17.2, −14.5)	−15.0 (−17.4, −12.6)	−0.87	0.5361	0.6938
T1	−24.9 (−26.3, −23.6)	−21.5 (−23.9, −19.1)	−3.42	0.0166*	0.1217
LVAP4pLS (%)					
T0	−16.2 (−17.5, −15.0)	−16.1 (−18.4, −13.9)	−0.10	0.9417	0.9997
T1	−19.6 (−20.9, −18.3)	−18.8 (−21.0, −16.5)	−0.81	0.5327	0.6938
RV_Basal (%)					
T0	−16.3 (−17.4, −15.2)	−14.9 (−16.5, −13.3)	−1.36	0.1830	0.3615
T1	−23.6 (−25.3, −22.0)	−20.8 (−23.3, −18.2)	−2.86	0.0665	0.2438
RV_Medial (%)					
T0	−14.3 (−15.3, −13.2)	−13.1 (−14.3, −11.9)	−1.16	0.1494	0.3615
T1	−20.7 (−22.1, −19.2)	−18.7 (−21.2, −16.3)	−1.93	0.1850	0.3615
RV_Apical (%)					
T0	−13.8 (−14.9, −12.7)	−12.2 (−13.8, −10.6)	−1.56	0.1255	0.3615
T1	−19.7 (−21.2, −18.2)	−17.8 (−19.9, −15.7)	−1.91	0.1565	0.3615
RVAP4pLS (%)					
T0	−14.5 (−15.5, −13.6)	−13.4 (−14.7, −12.1)	−1.11	0.1972	0.3615
T1	−18.6 (−19.9, −17.4)	−16.4 (−18.0, −14.9)	−2.19	0.0326*	0.1434

TGA, transposition of the great arteries; T0, fetal evaluation; T1, neonatal evaluation; LV, left ventricle; RV, right ventricle; Inter_V, interventricular septum; LVAP4pLS, left ventricle peak longitudinal strain from 4 chamber view; RVAP4pLS, right ventricle peak longitudinal strain from 4 chamber view. Estimated marginal means (EMMS) were estimated using a linear regression model including group, time with their interaction, adjusted for age, sex, gestational age at enrolment and gestational age at birth. Robust standard errors clustered by patient ID were used in the model.

a Adjusted p-values described pairwise comparisons in strain values between groups at each time point obtained from covariate-adjusted linear model and represent nominal (unadjusted for multiplicity) significance.

b *p*-corrected: *p*-value corrected for multiple comparisons using Benjamini-Hochberg procedure; 95% CI, 95% confidence interval.

* Significant result: *p* < 0.05.

**Figure 4:**
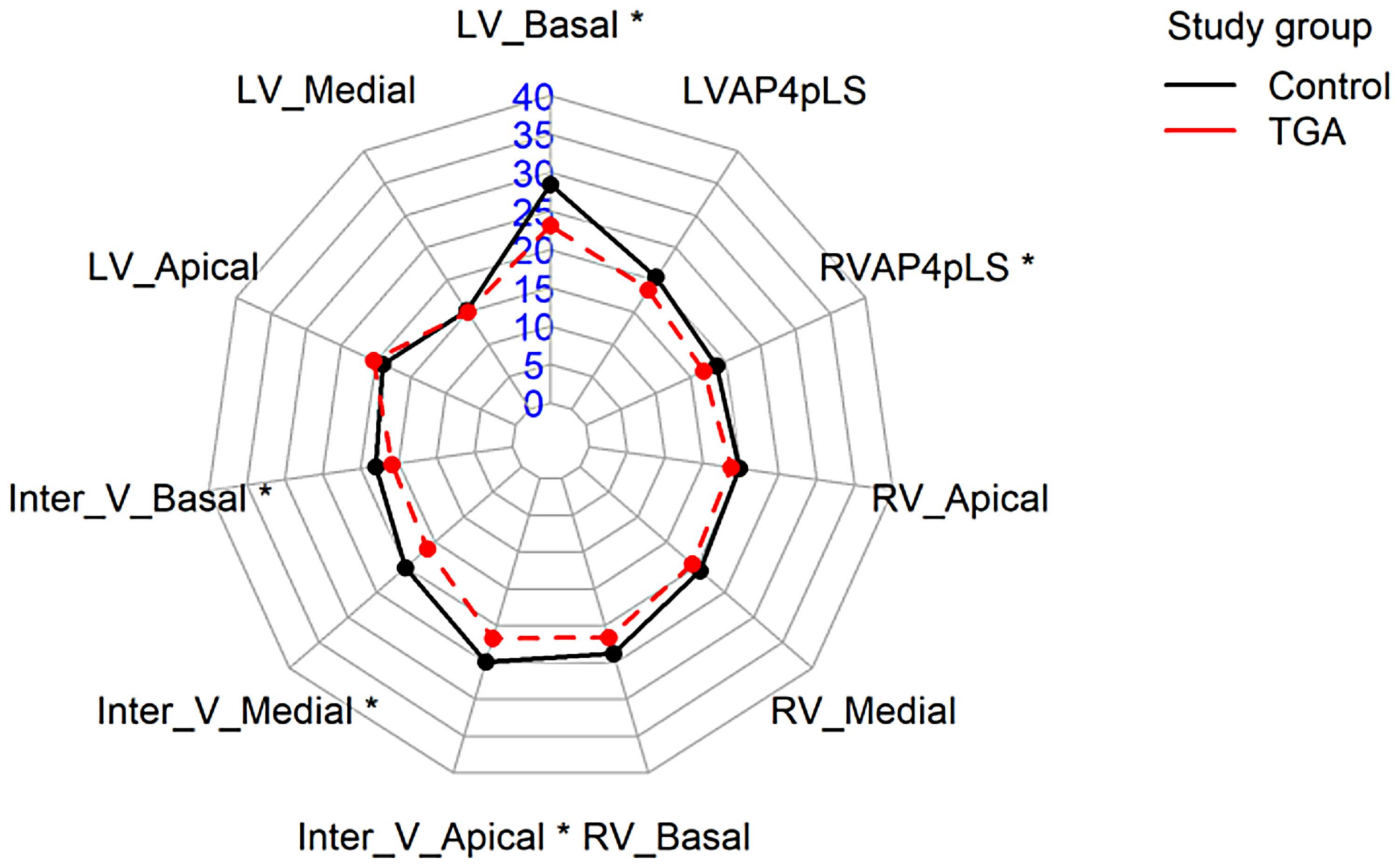
Radar chart showing differences in unadjusted means of absolute strain values (%) between TGA patients and controls. LV, left ventricle; RV, right ventricle; Inter_V, interventricular septum; LVAP4pLS, left ventricle peak longitudinal strain from 4 chamber view; RVAP4pLS, right ventricle peak longitudinal strain from 4 chamber view. An asterisk (*) denotes statistical significance for between-group differences based on nominal *p*-values.

Figure 4 showed differences in unadjusted mean values of segmental and global strain measurements between TGA patients and controls.

### 3.2 Differences in left ventricle, interventricular and right ventricle conventional measures

A significant group ⨯ time interaction effect was observed for LV_ESV (*p* = 0.009), RV_EDV (*p* < 0.0001) and RV_ESV (*p* = 0.005) indicating that between-group differences in mean values changed over time (Table 3).

**Table 3:** Differences in estimated marginal means (EMMS) of left ventricle and right conventional parameters between studied groups.

Time point	EMMS (95% CI) in Control group (*n*_1_ = 53)	EMMS (95% CI) in TGA group (*n*_2_ = 19)	Adjusted difference	*p*-value^a^
LV_EDV (mL)				
T0	2.04 (1.79, 2.29)	2.31 (1.99, 2.64)	−0.27	0.1802
T1	3.97 (3.58, 4.36)	3.44 (3.06, 3.82)	0.53	0.0540
LV_ESV (mL)				
T0	0.92 (0.79, 1.06)	1.11 (0.89, 1.32)	−0.19	0.1392
T1	1.92 (1.68, 2.16)	1.51 (1.29, 1.72)	0.41	0.0115*
LV_EF (%)				
T0	56.4 (54.2, 58.6)	56.8 (52.8, 60.7)	−0.38	0.8696
T1	53.1 (50.9, 55.3)	57.0 (53.0, 60.9)	−3.88	0.0934
RV_EDV (mL)				
T0	2.26 (1.98, 2.55)	3.03 (2.45, 3.61)	−0.77	0.0228*
T1	2.09 (1.93, 2.25)	4.48 (3.94, 5.01)	−2.38	<0.0001*
RV_ESV (mL)				
T0	1.33 (1.19, 1.41)	1.91 (1.53, 2.29)	−0.58	0.0084*
T1	1.30 (1.19, 1.41)	2.50 (2.25, 2.76)	−1.21	<0.0001*
RV_EF (%)				
T0	42.0 (39.5, 44.6)	38.9 (34.7, 43.2)	3.08	0.1994
T1	40.0 (37.9, 42.2)	42.6 (40.4, 44.8)	−2.59	0.1036

TGA, transposition of the great arteries; LV, left ventricle; RV, right ventricle; EDV, end-diastolic volume; ESV, end-systolic volume; EF, ejection fraction; T0, fetal evaluation; T1, neonatal evaluation; Estimated marginal means (EMMS) were estimated using a linear regression model including group, time with their interaction, adjusted for age, sex, gestational age at enrolment and gestational age at birth. Robust standard errors clustered by patient ID were used in the model.

a Adjusted p-values described pairwise comparisons in strain values between groups at each time point obtained from covariate-adjusted linear model and represent nominal (unadjusted for multiplicity) significance; 95% CI, 95% confidence interval.

* Aignificant result: *p* < 0.05.

### 3.3 Additional analysis in term fetuses and neonates with Tga

RVAP4pLS and RV_Medial showed significant differences between neonates with TGA who required Rashkind septostomy and those without Rashkind septostomy (Table 4). Although significant differences were noticed between the groups, neither strain parameter demonstrated significant discriminatory ability (for RV_Medial: unadjusted AUC = 0.66, 95% CI: 0.39–0.93, RVAP4pLS: unadjusted AUC = 0.73, 95% CI: 0.46–1).

**Table 4:** Differences in estimated marginal means (EMMS) of prenatal left ventricle, interventricular and right ventricle strain parameters between TGA neonates with Rashkind septostomy and without Rashkind septostomy.

Strain parameter	EMMS (95% CI) in TGA group without Rashkind septostomy (*n*_1_ = 7)	EMMS (95% CI) in TGA group with Rashkind septostomy (*n*_2_ = 12)	*p*-value^a^
LV_Basal (%)	−15.5 (−17.5, −13.5)	−16.6 (−18.1, −15.1)	0.362
LV_Medial (%)	−15.2 (−17.6, −12.9)	−17.1 (−18.9, −15.4)	0.215
LV_Apical (%)	−15.9 (−19.8, −12.0)	−16.0 (−18.9, −13.1)	0.943
Inter_V_Medial (%)	−15.4 (−18.5, −12.3)	−16.0 (−18.3, −13.7)	0.775
Inter_V_Apical (%)	−15.9 (−19.5, −12.4)	−14.7 (−17.3, −12.0)	0.571
LVAP4pLS (%)	−15.4 (−17.9, −12.9)	−16.1 (−18.0, −14.3)	0.643
RV_Basal (%)	−14.5 (−16.9, −12.1)	−16.1 (−17.9, −14.3)	0.286
RV_Medial (%)	−12.4 (−13.9, −11.0)	−14.5 (−15.6, −13.5)	0.031*
RV_Apical (%)	−11.5 (−13.5, −9.5)	−13.7 (−15.2, −12.2)	0.094
RVAP4pLS (%)	−11.7 (−13.5, −10.0)	−15.0 (−16.3, −13.7)	0.007*

TGA, transposition of the great arteries; LV, left ventricle; RV, right ventricle; EDV, end-diastolic volume; LVAP4pLS, left ventricle peak longitudinal strain from 4 chamber view; RVAP4pLS, right ventricle peak longitudinal strain from 4 chamber view. Estimated marginal means (EMMS) were derived from ANCOVA models including gestational age at enrolment as covariate.

a *p*-values from ANCOVA model and represent nominal (unadjusted for multiplicity) significance; 95% CI, 95% confidence interval.

* Significant result: *p* < 0.05.

In neonates with TGA, a significant interaction between inter_V_basal and gestational age at inclusion was observed (*p* = 0.0374) and adjusted main effects are not reported.

## 4 Discussion

This prospective study provides novel insights into myocardial deformation in TGA by evaluating the transition from fetal to early neonatal life. The main findings indicate preserved myocardial deformation in utero, followed by regional changes in myocardial deformation after birth, predominantly affecting the interventricular septum and right ventricle. These results highlight the importance of ventricular interdependence during perinatal adaptation. Previous prospective studies have described the structural, hemodynamic and conventional functional changes occurring during the fetal-to-neonatal transition in TGA. The present study extends these observations by providing a detailed assessment of regional myocardial deformation using speckle-tracking echocardiography. While conventional echocardiographic parameters characterize global ventricular adaptation, strain analysis offers complementary information regarding subtle regional myocardial mechanics, particularly at the level of the interventricular septum and right ventricle.

Demographic data showed no statistically significant differences between the two groups in terms of weight parameters in both fetuses and newborns (Table 1). In contrast, neonates with TGA showed impaired adaptation to extrauterine life, as reflected by lower APGAR scores. This finding is consistent with the hemodynamic alterations observed in neonates with TGA.

Speckle-tracking parameters were assessed for both ventricles in fetuses from the control and TGA groups. As shown in Table 2, no significant differences were observed between groups at baseline (fetal evaluation). The absence of significant differences in fetal strain parameters suggests that myocardial deformation may be relatively preserved under fetal circulatory conditions despite the abnormal cardiovascular physiology of TGA. In contrast, differences in septal and right ventricular deformation became apparent after birth as identified by nominal p-values, suggesting that the abrupt changes in ventricular loading associated with the transition to neonatal circulation may reveal regional mechanical differences that are not evident prenatally.

However, the absence of significant fetal differences should not be interpreted as evidence of prognostic value. The present study was not designed to evaluate the prognostic performance of fetal strain, and our findings provide no evidence that a single fetal strain measurement can predict postnatal clinical severity. The prognostic value of fetal myocardial deformation across the full clinical spectrum of TGA therefore remains undetermined.

In fetuses with TGA, oxygen saturation in the pulmonary artery is greater than 70%, compared to 55% in normal fetuses. Saturation in the ascending aorta and cerebral circulation is up to 20% lower than in normal fetuses. Both the fetal pulmonary circulation and the ductus arteriosus become increasingly sensitive to high oxygen content towards the end of gestation, which may predispose to foramen ovale obstruction, ductus arteriosus restriction at term and to redistribution of blood flow (9, 23–26). Moreover, higher oxygen saturation in the pulmonary artery leads to a decrease in pulmonary vascular resistance, an increase in pulmonary venous return and, respectively, an increase in LV preload (9, 24, 27).

Speckle-tracking parameters were assessed for both ventricles in newborns from both groups. Table 2 (T1) shows the results of this analysis. Notably, the absolute values of all three segmental parameters at the septum level are lower in the group of newborns with TGA, the difference between the two groups being nominally statistically significant. After Benjamini-Hochberg correction, only the medial interventricular septal strain remains statistically significant. In contrast, the LVAP4pLS parameter, which reflects global LV longitudinal deformation does not show statistically significant differences between the two groups. These findings indicate that the interventricular septum is particularly sensitive to perinatal changes in ventricular loading conditions, specific to the transition period in the case of neonates with TGA.

Regarding the RV, the segmental speckle tracking parameters do not show statistically significant differences between the two groups. In contrast, the speckle tracking parameter RVAP4pLS that reflects global RV longitudinal deformation has significantly lower absolute values in neonates with TGA based on nominal p-values. This likely reflects the fact that, in TGA, the RV is subjected to increased hemodynamic stress, functioning as a systemic ventricle since the fetal period. Previous studies have shown that the systemic RV presents progressive myocardial remodeling and changes in ventricular mechanics detectable by myocardial deformation techniques (28–30).

Lachaud et al. conducted a retrospective longitudinal study where they included fetuses with TGA from the time of diagnosis until the 38th week of gestation and compared them with healthy fetuses. They demonstrated that compared with normal fetuses, those with TGA exhibit a complex redistribution of blood flow in the second half of pregnancy, characterized by increased global pulmonary flow, reduced ductal flow (with negative diastolic flow towards the end of pregnancy), and restriction of the foramen ovale. In addition, fetal cardiac hemodynamic abnormalities have been associated with lower postnatal SO_2_ values (8).

In TGA, restriction of the PFO increases right atrial pressure and RV preload, while reduced PDA may increase RV afterload. These changes result in altered ventricular loading conditions and are consistent with our findings (Table 3), which show significantly increased RV volumes in TGA fetuses. This supports the concept of ventricular interaction and early remodeling in the systemic RV (31, 32).

Another observation of our study was the significant reduction in interventricular septal strain in the basal, medial, and apical segments in newborns with TGA compared with controls based on nominal p-values (after correction for multiple testing only medial interventricular septal strain showed a significant difference between groups).These results suggest that septal mechanics are particularly sensitive to changes in ventricular loading conditions characteristic of the pathophysiology of TGA. In this congenital malformation, the RV becomes the systemic ventricle and is exposed to increased afterload, while the LV functions in a circuit with relatively low pulmonary resistance. These differences in ventricular loading may determine changes in interventricular septal geometry and regional myocardial mechanics. Taken together, these findings may have clinical relevance, as early detection of septal reduced strain magnitude could improve functional assessment during the critical perinatal period.

The preservation of global LV longitudinal strain despite reduced septal strain magnitude may be explained by the regional nature of early myocardial adaptation. In TGA, the neonatal transition is characterized by abrupt changes in ventricular loading: the RV becomes exposed to systemic afterload, whereas the LV ejects into the pulmonary circulation. These altered loading conditions may primarily affect septal geometry and septal fiber shortening through ventricular interdependence, while free-wall LV deformation remains relatively preserved. Consequently, global LV strain may remain within a compensated range, masking regional reductions in septal strain magnitude. These findings support the concept that septal deformation may represent an early and load-sensitive marker of ventricular interaction during the perinatal transition in TGA.

In the additional analysis performed in newborns with TGA, we observed significant differences in RV strain parameters between patients who required Rashkind atrial septostomy and those who did not (Table 4). Although RVAP4pLS showed a point estimate for AUC of 0.73, this analysis should be interpreted as exploratory and underpowered. The relatively small sample size of our group resulted in wide 95% CI for the AUC estimate (95% CI: 0.46–1) which includes the null value of 0.50 indicating that the discriminative ability of RVAP4pLS cannot be considered statistically distinguishable from chance. The findings should be regarded as hypothesis-generating rather than confirmatory. As secondary outcomes, these parameters were not subject to correction for multiple testing. Larger cohorts are needed to confirm whether ventricular strain parameters could provide additional information regarding the severity of prenatal hemodynamic changes and the need for postnatal interventions.

### 4.1 Clinical implications

In this selected cohort, speckle-tracking echocardiography provided additional information on regional myocardial mechanics during the fetal-to-neonatal transition in TGA. The observed septal and right ventricular deformation differences -significant based on nominal p-values, though not all retained significance after correction for multiple testing, may reflect the sensitivity of myocardial strain to altered loading conditions and ventricular interdependence after birth. These findings should be interpreted as physiological observations rather than evidence of established myocardial dysfunction or of a role for fetal strain in perinatal risk stratification. The present study does not establish fetal strain as a predictor of postnatal severity or the need for intervention.

### 4.2 Study limitations

The present study has several limitations that must be taken into account when interpreting the results.

One limitation is the relatively small size of the group of patients with TGA. This is frequently encountered in studies of rare congenital heart defects. An additional limitation is the highly selected nature of the study population. The cohort included only fetuses with prenatally diagnosed simple TGA who underwent paired fetal and early neonatal echocardiographic examinations. Patients with severe pulmonary hypertension were excluded. Therefore, our findings are applicable primarily to a relatively stable subgroup of prenatally diagnosed simple TGA and should not be extrapolated to the entire clinical spectrum of TGA.

Another limitation of the present study is that healthy fetuses and neonates served as the only comparator group. Although this design allowed us to characterize differences from normal perinatal cardiovascular adaptation, it did not allow us to distinguish changes related to the underlying TGA anatomy from those associated with prenatal diagnosis, planned perinatal management, postnatal hypoxemia, or therapeutic interventions. In addition, our cohort did not allow comparisons between prenatally and postnatally diagnosed TGA, clinically stable and unstable neonatal presentations, or patients requiring urgent versus non-urgent balloon atrial septostomy. These questions deserve further investigation in larger prospective studies.

As stated in the study objectives, the conventional echocardiographic parameters and the need for Rashkind septostomy were considered secondary, exploratory outcomes. Accordingly, no formal adjustment for multiple testing was applied, which can increase the risk of type I error. Additional analysis of the prediction of the need for Rashkind atrial septostomy included a small number of patients, and RVAP4pLS did not demonstrate discriminative ability beyond chance. Therefore, the results should be regarded as exploratory and confirmed in future studies with larger sample sizes.

Speckle-tracking analysis also depends significantly on the quality of echocardiographic images. In our study, six patients were excluded due to insufficient image quality for strain analysis. Moreover, myocardial strain values are known to be vendor-dependent, reflecting differences in image acquisition, post-processing algorithms, and tracking methodologies.

A difference in gestational age at enrollment between the two studied groups represents a potential source of confounding. The difference in gestational age at enrolment between groups likely reflects routine clinical practice, as fetuses with TGA undergo serial fetal cardiology surveillance until delivery to monitor the risk of progressive foramen ovale restriction and to optimize perinatal management, whereas healthy fetuses do not routinely require specialized late-gestation cardiac follow-up. Although gestational age at enrolment and gestational age at birth were included as covariates in all regression models, residual confounding cannot be completely excluded given the developmental sensitivity of fetal strain parameters and the relatively small sample size. The inter-assessment time interval between evaluations differed significantly between groups highlighting differences in gestational age at enrollment. As both gestational age at inclusion and gestational age at birth were included as covariates in the regression models, the different follow-up time interval was implicitly accounted for, nevertheless, we cannot fully exclude the residual confounding. Therefore, the observed between-group differences should be interpreted with caution.

All speckle-tracking analyses were performed by a single experienced operator. Inter- and intra-observer reproducibility were not formally assessed using the fetal and neonatal images included in the present cohort, which represents a methodological limitation of the study. Therefore, the measurement reproducibility of the strain parameters obtained in the current dataset cannot be directly established.

Right ventricular volumetric assessment represents an additional methodological limitation. RV volumes were derived using the aCMQ algorithm, while the specific geometric assumptions underlying RV volume calculation are not explicitly provided to the operator, and validation of this approach specifically for fetal and neonatal right ventricular volumetry is limited. Furthermore, ventricular volumes were analyzed as absolute values and were not indexed to body surface area or another measure of body size. Consequently, differences in body and cardiac size may have influenced between-group comparisons, and the volumetric findings should be interpreted cautiously.

Finally, the present study did not evaluate coronary artery anatomy, surgical characteristics, postoperative ventricular function, perioperative complications, or long-term clinical outcomes. Consequently, the prognostic relevance in postoperative evaluation of the observed myocardial deformation abnormalities remains unknown and cannot be inferred from the present data.

### 4.3 Future directions

Future prospective longitudinal studies should include patients across the full spectrum of TGA severity and extend myocardial deformation assessment beyond a single fetal measurement. Serial evaluation during late gestation and analysis of fetal-to-neonatal changes in myocardial strain may help determine whether specific deformation patterns are associated with clinically relevant outcomes, including severe postnatal hypoxemia, urgent balloon atrial septostomy, preoperative hemodynamic instability, postoperative ventricular function, and intensive care requirements. Longer-term follow-up after arterial switch operation should further evaluate whether perinatal deformation patterns are associated with subsequent ventricular remodeling and functional outcomes. Such studies are needed to establish whether serial myocardial deformation assessment provides prognostic information beyond established anatomical and hemodynamic parameters.

## 5 Conclusions

In this cohort of prenatally diagnosed simple TGA, fetal myocardial deformation parameters did not differ significantly from those of healthy controls, whereas load-sensitive regional differences in interventricular septal and right ventricular deformation identified as nominal associations became apparent after birth. These findings suggest that the fetal-to-neonatal circulatory transition and the associated changes in ventricular loading may reveal regional mechanical differences that are not evident under fetal circulatory conditions, providing further insight into ventricular interdependence and early cardiovascular adaptation in TGA.

The present findings should be interpreted as physiological observations and do not establish fetal strain as a predictor of postnatal clinical severity. Future prospective longitudinal studies including the full spectrum of TGA severity and integrating serial fetal and fetal-to-neonatal strain assessment with clinically relevant early, postoperative, and longer-term outcomes are needed to determine the clinical and prognostic significance of these deformation patterns.

## Data Availability

The raw data supporting the conclusions of this article will be made available by the authors, without undue reservation.
